# The Role of Hyaluronic Acid for Soft Tissue Indications: A Systematic Review and Meta-Analysis

**DOI:** 10.1177/19417381211073316

**Published:** 2022-02-03

**Authors:** Moin Khan, Ajaykumar Shanmugaraj, Carlos Prada, Ashaka Patel, Eric Babins, Mohit Bhandari

**Affiliations:** † Division of Orthopaedic Surgery, Department of Surgery, McMaster University, Hamilton, Ontario, Canada; ‡ Department of Health Research Methods, Evidence, and Impact, McMaster University, Hamilton, Ontario, Canada; § Schulich School of Medicine, Western University, London, Ontario; ‖ University of Calgary, Calgary, Alberta, Canada

**Keywords:** hyaluronic acid, Injections, soft tissue injuries, randomized controlled trial (RCT), systematic review, meta-analysis

## Abstract

**Context:**

Soft tissue injuries are often treated with injectables such as corticosteroids and platelet-rich plasma (PRP) to reduce inflammation and promote healing. There is increasing evidence examining the use of hyaluronic acid (HA) for the management of soft tissue injuries.

**Objective:**

To evaluate the treatment effect and role of HA for available soft tissue indications.

**Data Sources:**

A search of PubMed, MEDLINE, EMBASE, and CENTRAL from the inception date of each database through February 24, 2021, was conducted for all randomized controlled trials (RCTs) involving the use of HA for soft tissue indications. Two reviewers independently screened articles for eligibility and extracted data from included studies for analysis. We assessed risk of bias for all included studies and pooled outcomes using a fixed-effects model. Outcomes (ie, function and pain relief) were categorized to short-term (<6 weeks, 6-12 weeks) and mid-term (>12 weeks) data. We present effect estimates as mean differences (MDs) and standardized mean differences (SMDs) and present the estimate of effect of HA for available indications in relation to available comparators.

**Study Design:**

Meta-analysis of RCTs.

**Level of Evidence:**

Level 1.

**Results:**

Of the 6930 articles screened, 19 RCTs (n = 1629 patients) were eligible and included in this review. HA was evaluated across a variety of soft tissue indications including rotator cuff disease, elbow pain, ankle sprains, Achilles tendinopathy, patellar tendinopathy, and trigger finger. Of the 19 RCTs, 11 were placebo-controlled and 9 used active comparators (PRP, cortisone, prolotherapy, or extracorporeal shockwave therapy). The pooled treatment effect of HA across most soft indications against placebo and active comparators demonstrated benefit in short-term pain <6 weeks (MD visual analogue scale [VAS] 2.48, 95% CI 2.31-2.65) and 6 to 12 weeks (MD VAS 2.03, 95% CI 1.86-2.20). Mid-term pain relief also favored HA over comparators across indications >12 weeks from administration (MD VAS 3.57, 95% CI 3.35-3.78). High heterogeneity was present with rotator cuff (10 trials, I^2^ = 94%), and elbow tendinopathy (2 trials, I^2^ = 99%). We identified uncertain benefit for trigger finger (2 trials, I^2^ = 67%). Heterogeneity for ankle sprains, patellar tendinopathy and Achilles tendinopathy could not be assessed as they only had 1 trial each.

**Conclusion:**

This systematic review and meta-analysis support HA’s efficacy in the treatment of a variety of soft tissue indications. Understanding the relative effects of HA to other injectable modalities requires additional, large trials.

Common soft tissue injuries among athletes and the general population include rotator cuff tendinopathy, elbow pain, Achilles and patellar tendinopathy, olecranon and pes anserine bursitis, and plantar fasciitis.^10^ Soft tissue musculoskeletal injury is one of the most common presenting complaints to primary care physicians and is estimated to account for over 50% of all musculoskeletal injuries reported in the United States annually.^31,32^ The diagnosis and management of such injuries represent a substantial financial burden, estimated at more than USD 15.8 billion annually.^25^

Treatments for soft tissue injuries include a wide range of therapeutic modalities, including oral analgesics, injections, physiotherapy, and surgery. Corticosteroid injections are used extensively because of low cost and efficacy in the short-term reduction of pain and improvement of function. However, recent research suggests that corticosteroid injections may be deleterious over long periods.^30,38^ Platelet-rich plasma (PRP) has been accumulating a growing body of evidence of support in the treatment of soft tissue injuries.^7^ As an autologous blood product, PRP therapy contains growth factors and other mediators that can promote healing of soft tissue injuries with long-lasting impact. While PRP therapy is considered safe, it is often costly and can take months before a clinical benefit is realized.^30^

There is increasing interest in the use of hyaluronic acid (HA), a naturally produced substance in the extracellular matrix of soft connective tissue and synovial fluid, for the management of soft tissue injuries given its various physiologic functions and properties.^35,39^ A number of clinical trials have demonstrated benefit with the use of HA injections for various indications.^3,14^ There have been several trials published recently evaluating soft tissue indications; however, controversy exists with regard to the effect, safety and relative efficacy of HA in comparison with other soft tissue injectable treatment options.

Understanding the efficacy of HA injection therapy can help guide patient and physician management of soft tissue injuries and improve care for patients with soft tissue injury. The purpose of this study is to systematically assess the literature to evaluate the role of HA in soft tissue musculoskeletal injuries and identify the relative efficacy of this intervention in comparison to other conservative and active interventions.

## Methods

This study was conducted according to the methods of the Cochrane Handbook and is reported according to the Preferred Reporting Items for Systematic Reviews and Meta-Analyses (PRISMA) statement.^8,22^

### Search Strategy

PubMed, EMBASE, MEDLINE, and Cochrane Central Register of Controlled Trials (CENTRAL) were searched for randomized controlled trials (RCTs) using HA injection therapy for soft tissue injuries from data inception to February 24, 2021. The search terms included *hyaluronic acid*, *injections*, *soft tissue*, *ligaments*, *tendons*, and similar phrases (Appendix 1, available in the online version of this article).

MeSH and EMTREE terms were used in various combinations and supplemented with free text to increase sensitivity. We consulted with experts in the field, manually reviewed the reference lists of articles that fulfilled the eligibility criteria and used the “related articles” feature in PubMed. Ongoing trials were identified from ClinicalTrials.gov.

### Eligibility Criteria

All RCTs related to soft tissue indications for HA were included in this systematic review. The research question and inclusion and exclusion criteria were established *a priori*. Inclusion criteria were (1) HA injection, (2) soft tissue injuries (eg, Achilles/hamstring tendinopathy, and rotator cuff tears), (3) nonsurgical studies, and (4) RCTs. No restriction was made regarding publication date, language, presence or absence of cointerventions, or length of follow-up. The exclusion criteria were (1) surgical studies, (2) non-RCTs, (3) review articles, (4) cadaver/nonhuman studies, and (5) non–soft tissue indications.

### Study Screening

All titles and abstracts were independently screened for eligibility by 2 reviewers with methodological and content expertise using a piloted electronic database^29^ (Excel, Microsoft Corp). All discrepancies were resolved by consensus. Duplicate articles were manually excluded. Both reviewers then reviewed the full text of all studies identified by title and abstract screening to determine final eligibility.

### Assessment of Risk of Bias

Two reviewers independently assessed the methodologic quality of all included studies. Risk of bias of included RCTs was assessed using the Cochrane Collaboration’s Risk of Bias tool.^36^ The Cochrane Risk of Bias tool evaluates studies in 5 domains of bias (ie, randomization, intended interventions, missing outcome data, measurement of the outcome, and selection of the reported result) as having high, some concerns, or low risk of bias. The reviewers resolved discrepancies by consensus.

### Extraction of Data

All data extraction was conducted using a standardized pilot-tested form. Data regarding study characteristics, patient demographics, treatments compared, and relevant outcomes were extracted. Any retrieved articles that were deemed to be reporting on the same patient population were included as a single study within the systematic review. If important data were unclear or not reported, attempts were made to contact the study authors for clarification. Critical outcomes were determined to be patient-important outcomes related to pain, function, and postintervention complications. Postintervention pain was assessed using a visual analogue scale (VAS), and functional outcomes were measured by disease-specific assessment scales.

### Statistical Analysis

Interobserver agreement for reviewers’ assessments of study eligibility was calculated with Cohen’s kappa (κ) coefficient. On the basis of the recommendations by Landis and Koch,^16^ a κ of 0 to 0.2 represents slight agreement; 0.21 to 0.40, fair agreement; 0.41 to 0.60, moderate agreement; and 0.61 to 0.80, substantial agreement. A value greater than 0.80 is considered almost complete agreement. Interobserver agreement for assessments of methodological quality was calculated with the intraclass correlation coefficient (ICC). The κ and ICC were calculated using SPSS software Version 28 (IBM Corp).

Descriptive statistics were calculated to reflect the frequency and percentage of abstracted study data, and results were pooled when possible. Continuous data were presented as mean differences (MDs) with a 95% CI as well as standardized mean differences (SMDs). We used SMDs to summarize outcome instruments that measured similar constructs. We pooled SMDs from individual trials to obtain the pooled estimate of effect for each outcome. When change scores were presented these were pooled in accordance with Cochrane guidelines.^8^ When standard deviations (SDs) were not available, they were calculated from alternative measures or were otherwise estimated from trials within the same comparison with similar scales, outcomes, and periods. When means were not available, we utilized median scores. We extracted data from graphical representations when required. We transformed scores when required to ensure that higher scores indicated improved function in all cases.^8^

Outcomes were dichotomized to short-term (<6 weeks, 6-12 weeks) and mid-term (>12 weeks). When multiple comparators were present, we prioritized those of placebo interventions when pooling data. Primary outcome for this study was VAS for pain in the short-term after intervention. Complications were tabulated and presented descriptively. Pooled data were analyzed using a fixed-effect meta-analysis using the inverse-variance method given the assumption that all effect estimates for HA efficacy estimate the same underlying intervention effect. Findings were evaluated with regard to clinical importance on the basis of the minimal clinically important difference (MCID), which is the smallest difference that a patient may find beneficial.^33^ A commonly utilized estimate of the MCID is 0.5 times the SD of a sample. For the purposes of this review, we used this threshold for clinical importance.^23^

To assess for publication bias, we constructed funnel plots that examined sample size versus exposure effect across included trials for outcomes at 6-week follow-up (Appendix 2, available online). The forest and funnel plots were created with RevMan 5.4 (The Cochrane Collaboration).

### Evaluation of Heterogeneity and Sensitivity Analysis

The χ^2^ and I^2^ statistics were used to measure the heterogeneity of results within the included studies. For the χ^2^ test, a *P* < 0.05 was considered significant. The I^2^ test was categorized as follows: 0.0% to 24.9% indicating no heterogeneity, 25.0% to 49.9% indicating low heterogeneity; 50.0% to 74.9% indicating moderate heterogeneity; 75.0% to 100.0% indicating high heterogeneity. We developed a priori hypotheses to explore both potential artifactual and real differences of treatment effect across trials. Subgroup analysis was planned a priori by intervention. Sensitivity analyses were planned for studies to investigate the effects of missing data.

## Results

### Search Results and Study Characteristics

**Figure 1.**
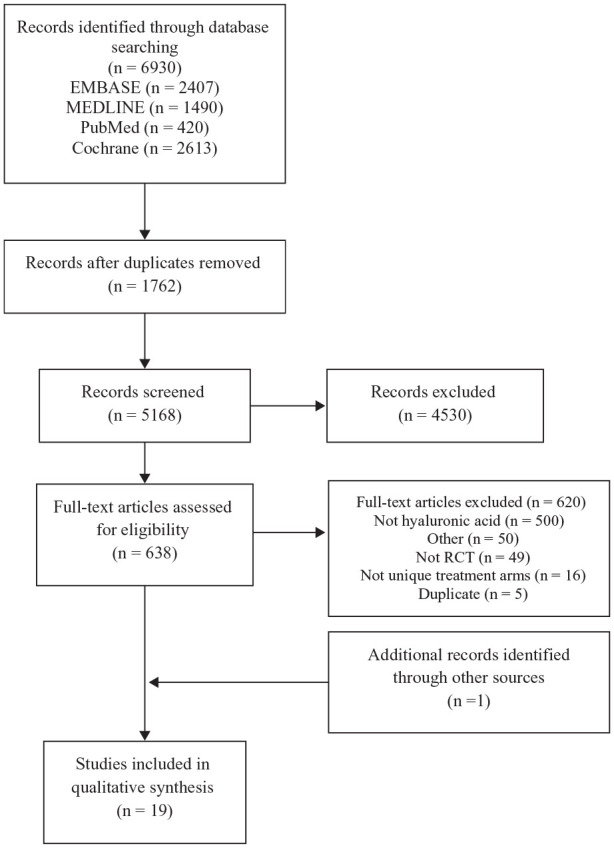
Preferred Reporting Items for Systematic Reviews and Meta-Analyses (PRISMA) flow diagram. RCT, randomized controlled trial.

The search result identified 6930 potentially relevant studies. After the application of inclusion and exclusion criteria and removal of duplicates, 638 studies underwent full text review. Nineteen RCTs were eligible for inclusion in this systematic review (Figure 1).^1-3,5,6,9,11-14,17,18,20,21,24,26-28,31,34^ There was substantial agreement between the 2 reviewers at the title/abstract screening stage (κ = 0.77; 95% CI 0.74-0.80) and almost complete agreement at the full-text screening stage (κ = 0.82; 95% CI 0.73-0.92).

Of the 19 trials published between 2001 to 2021, 9 (47.4%) were performed in Europe, 2 (10.5%) in North America,^27,28^ and the remaining 8 (42.1%), in Asia. All eligible trials included patients who were treated by HA injections for soft tissue indications with comparators, including cortisone, placebo, PRP, shockwave treatment, physiotherapy as well as prolotherapy. Sample sizes ranged from 24 to 331, and the total sample included 1629 patients. The mean age of patients was 48.5 (±9.9) years. Of the included trials, 11 (57.9%) evaluated HA injections versus comparator for soft tissue indications around the shoulder, 2 evaluated soft tissue indications around the ankle, elbow, and hand each, and 1 evaluated soft tissue injuries to the patellar tendon and Achilles tendon each (Table 1).

**Table 1.** Characteristics of included studies

	Publication Year	Location	Injury	Treatment Arms Description	Patients, n (% Male)		Age, y (Mean ± SD)		
Study					Intervention	Control	Intervention	Control	Risk of Bias^*a*^
Rezasoltani et al	2021	Iran	Rotator cuff disease	1. HA 2. Physiotherapy	28 (32.1)	23 (47.8)	47.07 ± 13.23	55.52 ± 8.16	Some concerns
Kanchanathepsak et al	2020	Thailand	Trigger finger	1. HA 2. Triamcinolone acetate	33 (21.2)	33 (27.3)	58.3 ± 11.2	54.7 ± 11	Some concerns
Apaydin et al	2020	Turkey	Lateral epicondylitis	1. HA 2. Dextrose prolotherapy	16 (18.8)	16 (18.8)	45.6 ± 4.7	43.3 ± 7.4	Some concerns
Kaux et al	2019	Belgium	Patellar tendinopathy	1. HA 2. PRP	15 (100)	18 (100)	29.3 ± 8.1	29.5 ± 9.9	High
Huang et al	2018	Taiwan	Hemiplegic shoulder pain	1. HA 2. Saline	18 (61.1)	9 (66.7)	59.7 ± 10.6	62 ± 9.3	High
Cai et al	2018	China	Rotator cuff disease	1. HA 2. Saline 3. PRP 4. PRP + HA	44 (54.5)	Saline: 47 (57.4) PRP: 45 (48.9) HA + PRP: 48 (54.2)	38.93 ± 7.35	Saline: 39.87 ± 8.96 PRP: 40.56 ± 7.85 HA + PRP: 39.63 ± 7.65	Some concerns
Frizziero et al	2017	UK	Rotator cuff disease	1. HA 2. ESWT	17 (23.5)	17 (23.5)	58.2	58.5	High
Rey et al	2017	Spain	Rotator cuff disease	1. HA + physiotherapy 2. Physiotherapy	42 (57.1)	42 (47.6)	42.3 ± 11.5	38 ± 13.1	High
Lynen et al	2016	Belgium	Achilles tendinopathy	1. HA 2. ESWT	40 (37.5)	40 (32.5)	45.8 ± 10	44.8 ± 9.6	Some concerns
Liu et al	2015	Taiwan	Trigger finger	1. HA 2. Triamcinolone acetonide	19 (15.8)	17 (17.6)	60.3 ± 13.6	65.8 ± 11.8	Low
Jakobs et al	2015	Germany	Ankle sprain	1. HA + standard care (eg, brace, crutches, RICE 2. Standard care	20 (60.0)	20 (75.0)	36.5 (range, 16-64)	25.5 (range, 17-73)	High
Penning et al	2014	Netherlands	Rotator cuff disease	1. HA + lidocaine 2. Traimcinolone acetonide + lidocaine 3. Saline + lidocaine	51	Triamcinolone acetonide + lidocaine: 53 Saline: 55	53 (range, 20-87)^*b*^	53 (range, 20-87)^*b*^	Low
Kim et al	2012	South Korea	Rotator cuff disease	1. HA 2. Dexamethasone disodium phosphate + lidocaine + saline	52 (21.2)	53 (32.1)	55.9 ± 7.9	54.1 ± 7.7	Some concerns
Chou et al	2010	Taiwan	Rotator cuff disease	1. HA 2. Saline	25 (36.0)	26 (38.5)	51.16 ± 7.84	52.38 ± 8.95	Some concerns
Petrella et al	2010	Canada	Lateral epicondylitis	1. HA 2. Saline	165 (55.2)	166 (53.0)	49 ± 15	47 ± 11	Some concerns
Özgen et al	2010	Turkey	Rotator cuff disease	1. HA 2. Physiotherapy	12 (25.0)	12 (25.0)	58.67 ± 9.8	52.5 ± 8.83	High
Meloni et al	2008	Italy	Rotator cuff disease	1. HA + lidocaine + saline 2. Saline + lidocaine	28	28	Range 31-71^*b*^	Range 31-71^*b*^	High
Petrella et al	2007	Canada	Ankle sprains	1. HA + standard care (eg, RICE) 2. saline + standard care	79	79	NR	NR	Low
Shibata et al	2001	Japan	Rotator cuff disease	1. HA + lidocaine 2. Dexamethasone + lidocaine	38 (71.0)	40 (70.0)	59.5 ± 9.1	62.4 ± 8.6	High

ESWT, extracorporeal shock wave therapy; HA, hyaluronic acid; NR, not reported; PRP, platelet-rich plasma; RICE, rest, ice, compression, and elevation.

a Risk of bias assessed using the Cochrane Risk of Bias Tool

b Not stratified by group.

### Risk of Bias

Of the included studies risk of bias assessment indicated only 3 of 19 trials to be of low risk of bias. Meanwhile, 8 had some concerns or were at high risk of bias. Agreement between reviewers in the assessment of risk of bias was high (ICC = 0.87, 95% CI 0.66-0.95).

### Outcomes

#### HA Across All Outcomes

**Figure 2.**
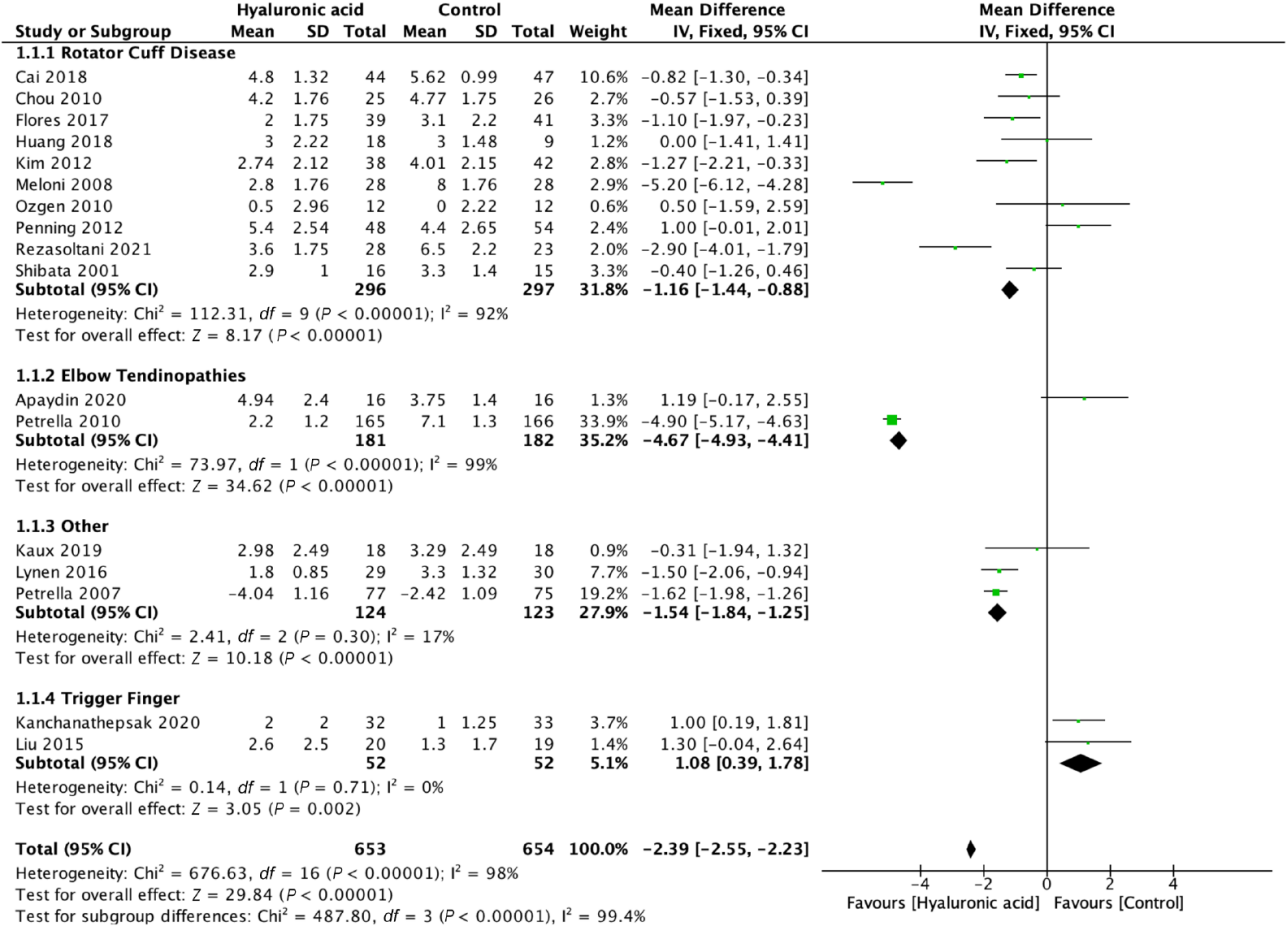
Pain ≤6 weeks, mean difference, visual analogue scale score.

**Figure 3.**
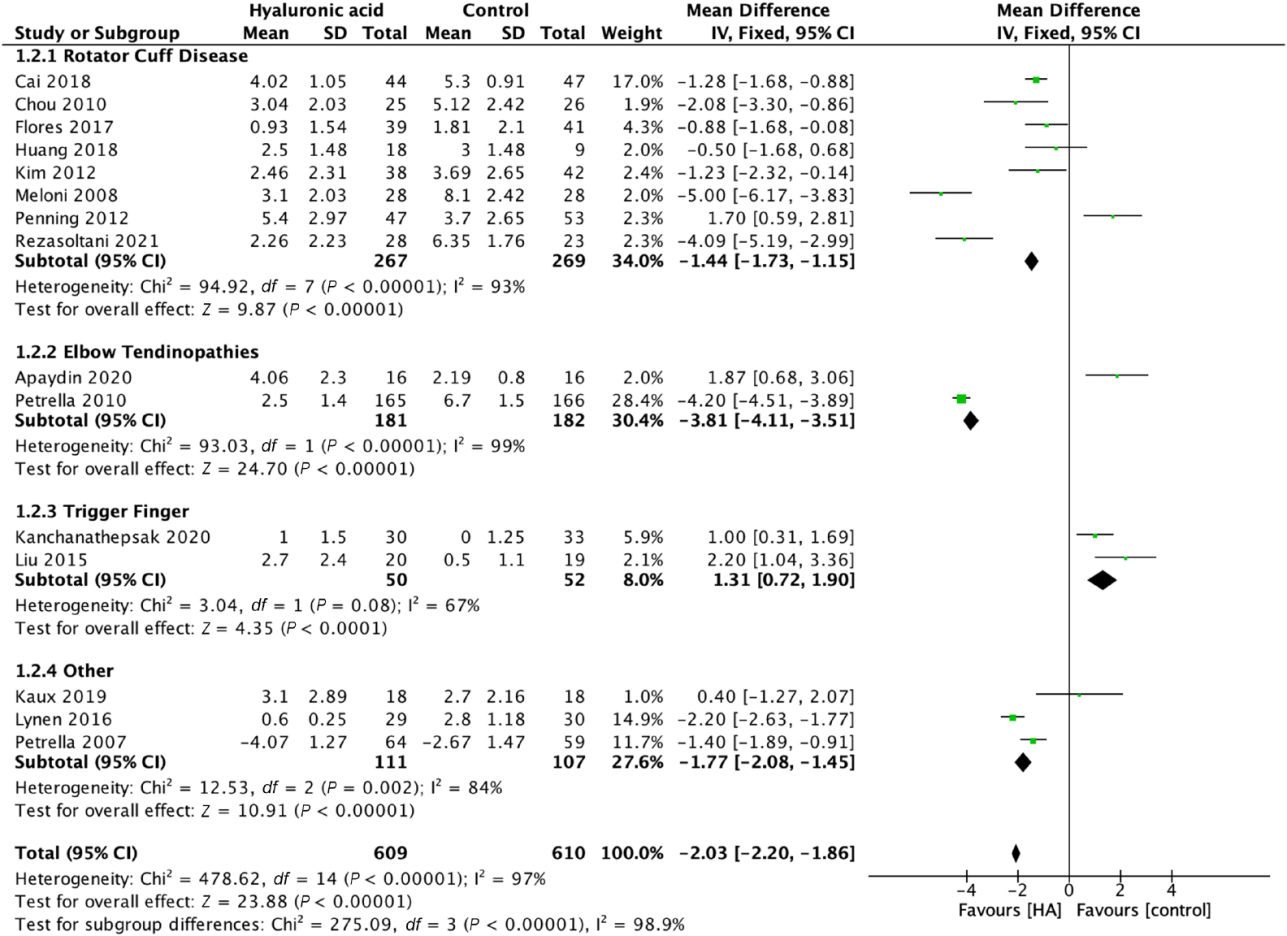
Pain >6 weeks, ≤12 weeks, mean difference, visual analogue scale score. HA, Hyaluronic acid.

We evaluated pain, function, and adverse events across all trials that reported them (17 RCTs, 1307 patients). Across all indications HA injections resulted in significant benefit with respect to pain at the short- and mid-term. Early results less than 6 weeks from administration favored HA injections over comparators in MD of self-assessed pain VAS (MD 2.39, 95% CI 2.23-2.55, *P* < 0.001; 17 trials, 1307 patients) (Figure 2), and from 6 to 12 weeks (VAS MD 2.03, 95% CI 1.86-2.20, *P* < 0.001; 15 trials, 1219 patients) (Figure 3). Mid-term outcomes with regard to pain relief also favored HA injections over comparators across indications >12 weeks from administration (VAS MD 3.57, 95% CI 3.35-3.78, *P* < 0.001; (6 trials, 656 patients) (Appendix 3, available online).

With regard to functional outcomes, as assessed by available pooled functional outcome scores (Constant and ASES [American Shoulder and Elbow Surgeons]), early results less than 6 weeks after administration of HA injections over comparator favored HA (SMD 0.30, 95% CI 0.07-0.52, *P* = 0.01; 4 trials, 324 patients) (Appendix 4, available online).

No serious adverse events were reported by any included trial. Patients rarely reported pain with injection. Lynen et al^18^ reported 3 nonserious adverse events, including moderate tendon pain after injection, Kim et al^14^ reported 5 patients, Flores et al^5^ reported 5, and Petrella et al^27^ reported 3 patients with pain either during or after injection. Two trials did not report complications.^6,24^

**Figure 4.**
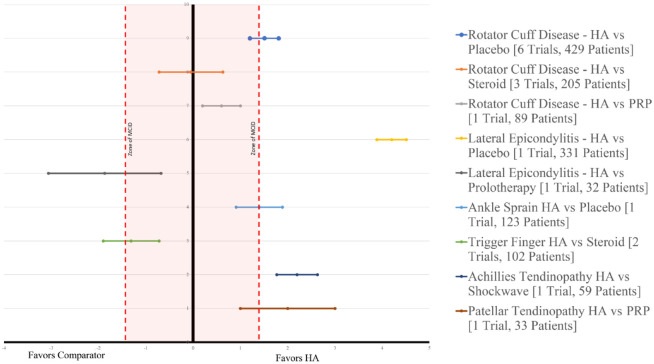
Effect of hyaluronic acid (HA) on pain relief by indication and comparator visual analogue scale scores (>6 weeks up to 12 weeks). PRP, platelet-rich plasma.

Heterogeneity identified in the overall pooled analyses was explored for cause. We evaluated both the type of comparator (placebo/active comparator) and indication for HA treatment as potential targets a priori for examination. Heterogeneity across all indications was high at ≤6 weeks (I^2^ = 98%), 6 to 12 weeks (I^2^ = 98.9%), and >12 weeks (I^2^ = 98.9%). Across all indications when HA injections were compared with placebo or standard physical therapy, a significant benefit was identified for HA as measured by pain reduction (VAS MD 2.91, 95% CI 2.73-3.10,*P* < 0.001 at ≤6 weeks with I^2^ = 98%) (Appendix 5, available online). Findings at 6 to 12 weeks were similar with VAS MD 1.51, 95% CI 1.20-1.81, *P* < 0.001 with substantial heterogeneity (I^2^ = 98%). Detailed breakdown of available evidence by comparator and indication present (Table 2, Figure 4).

**Table 2.** Effect of hyaluronic acid on pain relief by indication and comparator (>6 weeks up to 12 weeks)

	Comparator				
	Placebo ± Nonoperative Therapy	Steroid	Platelet-Rich Plasma	Shockwave	Prolotherapy
Indication	Effect Estimate, Mean Difference (MD) Visual Analogue Scale (0-10)				
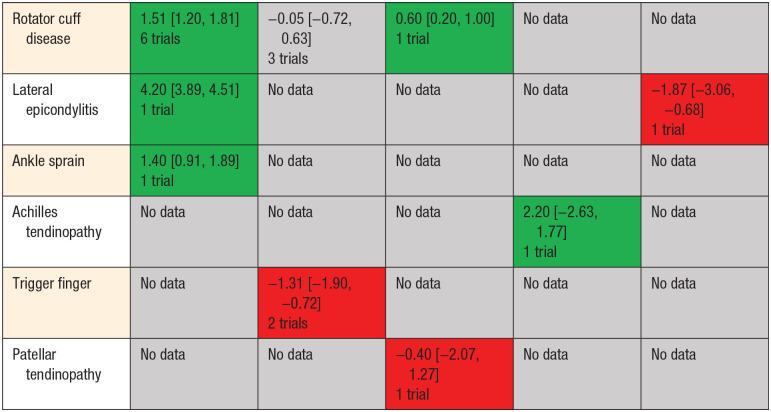					

Green, data supportive; Grey, inconclusive/ no data/; Red, data not supportive.

#### Treatment Effectiveness Across Indications

##### Shoulder

Of the 11 trials evaluating shoulder indications 10 were indicated for various stages of rotator cuff disease. This included the spectrum of conditions ranging from shoulder impingement, rotator cuff tendinopathy as well as partial thickness rotator cuff tears. Comparators include placebo (7 trials), steroid injections (3 trials), and PRP (1 trial). One trial evaluated the roll of subacromial HA injections compared with placebo in hemiplegic patients complaining of shoulder pain^9^ (Figure 3). Across all shoulder indications and comparators, HA injections resulted in an improvement: pain VAS MD 1.16, 95% CI 0.88-1.44, *P* < 0.001 (10 trials) (I^2^ = 92%). At <6 weeks and at 6 to 12 weeks postintervention VAS MD 1.44, 95% CI 1.15-1.73, *P* < 0.001 (8 trials) (I^2^ = 93%). Assessing only rotator cuff disease HA injections provided significant improvement in VAS score at <6 weeks: VAS MD 1.21, 95% CI 0.92-1.49, *P* < 0.001, I^2^ = 93% as well at the 6- to 12-week postintervention VAS MD 1.50, 95% CI 1.20-1.79, *P* < 0.001, I^2^ = 94%. When compared with saline or physical therapy alone HA injections resulted in significant improvement from 6 to 12 weeks postintervention: MD VAS 1.51, 95% CI 1.20-1.81, *P* < 0.001, I^2^ = 95%.

Functional outcomes as assessed by Constant score favored HA injections over comparators <6 weeks (MD 5.86, 95% CI 4.38-7.33, *P* = 0.001) (Appendix 6, available online) and from 6 to 12 weeks (MD 13.45, 95% CI 12.29-14.61, *P* < 0.001; 4 trials) (Appendix 7, available online).

##### Elbow

Lateral elbow pain was evaluated in 2 trials.^1,27^ Petrella et al^27^ compared HA injections with placebo and Apaydin et al^1^ evaluated HA injections versus prolotherapy. Pooled analysis found HA injections to provide significant benefit with respect to pain relief at <6 weeks: VAS MD 4.67, 95% CI 4.41-4.93, *P* < 0.001 as well as at 6 to 12 weeks: VAS MD 3.81, 95% CI 3.51-4.11, *P* < 0.001. Petrella et al^27^ reported improvements in mean VAS scores after grip strength testing favoring HA (2.0 vs 9.9).

##### Trigger finger

Both Kanchanathepsak et al^12^ and Liu et al^17^ evaluated the role of HA injections versus steroid injection for trigger finger. Pooled analysis found steroid performed better than HA with regard to pain at less than 6 weeks: VAS MD 1.08, 95% CI 0.39-1.78, *P* = 0.002, I^2^ = 0% and at 6 to 12-weeks postintervention: VAS MD 1.31, 95% CI 0.72-1.90, *P* < 0.001, I^2^ = 67%.^12,17^

##### Ankle sprains

Jakobs et al^11^ and Petrella et al^28^ evaluated the role of HA versus physical therapy in ankle sprains. Petrella et al^28^ identified a significant reduction in VAS pain on both weightbearing and walking was observed at day 8 for HA compared with placebo (5.6 vs 4.2, *P* < 0.05). Jakobs et al^11^ found periarticular injection of HA for ankle sprain resulted in significant (*P* < 0.05) earlier return to sports (23 vs 32 days) and relief in pain when walking and at rest.^11^

##### Patellar and Achilles tendinopathies

Kaux et al^13^ evaluated patellar tendinopathy comparing HA injections versus PRP. Similar findings between groups were reported with 14 out of 18 subjects (77.8%) in the PRP group and 11 out of 15 subjects (73.3%) in the HA group were responders to the treatments.^13^ Lynen et al^18^ evaluated HA injections versus extracorporeal shockwave therapy for Achilles tendinopathy and found a significantly greater benefit with HA with regard to pain relief in comparison to extracorporeal shockwave therapy with a decrease of 68.1% versus 47.9% at 4 weeks as well as decreases at the 3 and 6 month assessment points (*P* = 0.0030).

### Sensitivity Analysis

Sensitivity analysis found robust findings for analysis in which medians were utilized when means were unavailable: VAS MD 2.83 [3.02-2.65] versus 2.48 [2.65-2.31].^9,12,18,24^ Additionally, we performed sensitivity analysis for missing SDs requiring calculations from alternative measures or estimations from trials within the same comparison.^5,9,12,13,18,20,24,31^ Results of this analysis also found our findings to be robust and conservative VAS MD 2.85 [3.05-2.65] versus 2.48 [2.65-2.31].

## Discussion

This meta-analysis suggests that treatment effect estimate from HA, as a class, is effective for soft tissue indications for reducing pain and improving function. These findings are based on available RCTs providing the best evidence currently available with respect to the use of HA in this indication. Specifically, evidence suggests that (1) HA injections significantly reduced pain after pooled analysis in ankle sprains (2 trials), elbow pain (2 trials), and rotator cuff disease (8 trials); (2) the relative efficacy of HA injections versus other injectable modalities remains unclear and requires further trials; and (3) HA injections do not increase risk of serious adverse events.

When evaluating the function of HA injections by indication, we identified support for HA for rotator cuff disease, elbow pain, ankle sprains, as well as Achilles and patellar tendinopathy. Overall, the largest body of evidence available relates to HA injections in rotator cuff disease including partial thickness rotator cuff tears as well as shoulder impingement syndrome. We identified 6 trials comparing HA to placebo or standard of care treatments with pooled MD scores on VAS favoring HA by 1.51 (95% CI 1.20-1.81) points on a scale of 10 when assessed at 6 to 12 weeks after intervention. This score surpasses the MID (minimal important difference) for rotator cuff disease, which was identified by Tashjian et al^37^ as 1.4 on a scale of 10. The MID or MCID is the smallest effect that a patient who was informed regarding available treatment options would perceive as valuable enough to justify a change in therapeutic management when weighing the anticipated benefits against the possible harms of an intervention.^33^ Although HA demonstrated significantly improved pain in tendinopathies, the single trials for each of Achilles tendon and patellar tendon studies were small and insufficient for definitive conclusions.

While HA was not superior to steroid, based on pooled analysis from 3 trials, we did identify support from 1 trial for HA formulations over PRP in reducing pain from rotator cuff disease. Studies reporting Constant score for shoulder indications were also pooled and demonstrated support for functional improvement with HA over comparator at timepoints less than 6 weeks and 6 to 12 weeks postintervention. Findings at our primary endpoint at 6 to 12 weeks postintervention were MD in Constant score of 13.45. While the MCID for shoulder Constant score has a wide range of values reported in the literature from 8 to 36 points, the pooled results from this analysis exceed many of those reported values as well as the value identified by Kukkonen et al^15^ of 10.4 points as the threshold score in patients with rotator cuff pathology.^4^ Considering the concerns that exist on rotator cuff integrity with the repetitive use of steroid injections and the potential costs associated with PRP injections, these findings suggest HA injections as an appealing option to treat patients with rotator cuff disease that fails first-line treatment with analgesics and physiotherapy.^19^

Regarding safety of the assessed intervention, HA injections proved very safe at the short- and mid-terms with very few minor adverse events (<2.5% of transient tenderness at the site of injection) and no serious adverse events were reported. Studies assessing the safety of this intervention and its potential effects on tendon status in the long term are still needed but given the mechanism of action and pharmacology detrimental effects are unlikely.

### Limitations

When data were unavailable despite attempts to contact the authors, we estimated SDs based on similar studies or utilized other described methods to obtain estimates of SD. A sensitivity analysis confirmed that this was unlikely to change the results of our study. Another limitation is that the majority of the included studies were of small sample sizes and for some indications there was only 1 or 2 RCTs providing information. Thus, assessments for certain indications at various follow-up periods were not possible. Additionally, we identified a significant degree of heterogeneity across most outcomes despite controlling for indication and comparator, thus limiting our ability to perform subgroup analyses on HA versus other injection types. This supports the need for large high-quality studies to provide reliable estimates of effect for HA across various soft tissue indications. This also serves the need to determine the influence of the amount and type of HA used on patient outcomes. Last, there was limited use of various outcome measures across included studies, thus limiting our ability to accurately assess patient function and pain levels postintervention.

Another limitation was the fact that there was variability in both the number and dosage of the HA injections used in the intervention groups of the different trials; thus, recommendations on the type of HA and proper way to administer this product are still needed.

## Conclusion

This systematic review and meta-analysis supports the use of HA for several soft tissue indications. Future large trials are required to confirm effect size and indications as well as relative efficacy against commonly used comparators.

## Supplemental Material

sj-pdf-1-sph-10.1177_19417381211073316 – Supplemental material for The Role of Hyaluronic Acid for Soft Tissue Indications: A Systematic Review and Meta-Analysis Click here for additional data file. Supplemental material, sj-pdf-1-sph-10.1177_19417381211073316 for The Role of Hyaluronic Acid for Soft Tissue Indications: A Systematic Review and Meta-Analysis by Moin Khan, Ajaykumar Shanmugaraj, Carlos Prada, Ashaka Patel, Eric Babins and Mohit Bhandari in Sports Health: A Multidisciplinary Approach
