# Supplementary material for: The Role of Hyaluronic Acid for Soft Tissue Indications: A Systematic Review and Meta-Analysis
Source: Sports Health. 2022 Feb 3;15(1):86–96. doi: 10.1177/19417381211073316 (PMC9808833; doi:10.1177/19417381211073316)

ONLINE APPENDICES

Appendix 1. Search Strategy

**MEDLINE: 1490 studies**	**EMBASE: 2407 studies**	**PUBMED: 420 studies**	**COCHRANE: 2613 studies**
Strategy:<br>1. exp Hyaluronic Acid/ or hyal*.ti,ab. or HA.ti,ab.<br>2. platelet rich plasma.ti,ab. or exp Platelet-Rich Plasma/ or PRP.ti,ab.<br>3. exp Cortisone/ or cortisone.ti,ab. or corticosteroid.ti,ab. or exp Glucocorticoid/ or glucocorticoid.ti,ab. or kenalog.ti,ab. or triamcinolone.ti,ab. or tramcinolone.ti,ab. or depomedrol.ti,ab. or betamethasone.ti,ab. or methylprednisolone.ti,ab. or aristospan.ti,ab. or celstone.ti,ab. or corticosteroid shot.ti,ab. or exp Triamcinolone Acetonide/ or exp Methylprednisolone/ or exp Betamethasone/<br>4. (viscosup* or viskosup*).ti,ab.<br>5. orthovisc*.ti,ab.<br>6. synvisc*.ti,ab.<br>7. monovisc*.ti,ab.	Strategy:<br>1. exp Hyaluronic Acid/ or hyal*.ti,ab. or HA.ti,ab.<br>2. platelet rich plasma.ti,ab. or exp Platelet-Rich Plasma/ or PRP.ti,ab.<br>3. ortisone.ti,ab. or exp cortisone/ or glucocorticoid.ti,ab. or exp glucocorticoid/ or corticosteroid.ti,ab. or kenalog.ti,ab. or cortisone shot.ti,ab. or triamcinolone.ti,ab. or tramcinolone.ti,ab. or betamethasone.ti,ab. or depomedrol.ti,ab. or aristospan.ti,ab. or celestone.ti,ab. or methylprednisolone.ti,ab. or exp methylprednisolone acetate/ or exp triamcinolone/ or exp triamcinolone acetonide/ or exp triamcinolone hexacetonide/ or exp betamethasone/<br>4. (viscosup* or viskosup*).ti,ab.<br>5. orthovisc*.ti,ab.	Strategy:<br>Search: ((((synvisc OR orthovisc OR monovisc OR durolane OR viscosupplementation OR viskosupplementation OR hyaluronic acid OR HA OR hylan OR hyaluron OR platelet-rich plasma OR PRP OR cortisone OR glucocorticoid OR kenalog OR cortisone shot OR triamcinolone or tramcinolone OR depomedrol OR betamethasone OR methylprednisolone OR aristospan OR celestone OR corticosteroid) AND ((((((randomly OR random OR randomized OR trial OR compare OR comparative OR versus) OR ("clinical trial"[Publication Type])) OR ("controlled clinical trial"[Publication	Strategy:<br>(bursitis OR impingement or tendinopathy OR contusions OR tear OR rupture OR strains OR sprains OR Ligaments OR tendons OR muscle OR soft tissue OR knee OR ankle OR foot OR shoulder OR hip) AND (injection) AND (synvisc OR orthovisc OR monovisc OR durolane OR viscosupplementation OR viskosupplementation OR hyaluronic acid OR HA OR hylan OR hyaluron OR platelet-rich plasma OR PRP OR cortisone OR glucocorticoid OR kenalog OR cortisone shot OR triamcinolone or tramcinolone OR depomedrol OR betamethasone OR methylprednisolone OR aristospan OR

8. 1 or 2 or 3 or 4 or 5 or 6 or 7<br><br>9. inject*.ti,ab.<br><br>10. 8 and 9<br><br>11. clinical trial.ti,ab. or exp Clinical Trial/<br><br>12. controlled clinical trial.ti,ab. or exp Controlled Clinical Trial/<br><br>13. randomized controlled trial.ti,ab. or exp Randomized Controlled Trial/<br><br>14. exp Randomized Controlled Trials as Topic/ or random*.ti,ab.<br><br>15. clinical trials as topic.mp. or exp Clinical Trials as Topic/<br><br>16. placebo.ti,ab.<br><br>17. trial*.ti,ab.<br><br>18. Compar*.ti,ab.<br><br>19. versus.ti,ab.<br><br>20. 11 or 12 or 13 or 14 or 15 or 16 or 17 or 18 or 19<br><br>21. exp Ligaments/ or ligament*.ti,ab.<br><br>22. exp Tendons/ or tendon*.ti,ab.<br><br>23. muscle*.ti,ab. or exp Muscles/	6. synvisc*.ti,ab.<br><br>7. monovisc.ti,ab.<br><br>8. 1 or 2 or 3 or 4 or 5 or 6 or 7<br><br>9. inject*.ti,ab.<br><br>10. 8 and 9<br><br>11. clinical trial.ti,ab. or exp Clinical Trial/<br><br>12. controlled clinical trial.ti,ab. or exp Controlled Clinical Trial/<br><br>13. randomized controlled trial.ti,ab. or exp Randomized Controlled Trial/<br><br>14. exp Randomized Controlled Trials as Topic/ or random*.ti,ab.<br><br>15. clinical trials as topic.mp. or exp Clinical Trials as Topic/<br><br>16. placebo.ti,ab.<br><br>17. trial*.ti,ab.<br><br>18. compar*.ti,ab.<br><br>19. versus.ti,ab.<br><br>20. 11 or 12 or 13 or 14 or 15 or 16 or 17 or 18 or 19<br><br>21. exp Ligament/ or ligament*.ti,ab.	Type])) OR ("equivalence trial"[Publication Type])) OR ("pragmatic clinical trial"[Publication Type])) OR ("randomized controlled trial"[Publication Type]))) AND (bursitis OR impingement or tendinopathy OR contusions OR tear OR rupture OR strains OR sprains OR Ligaments OR tendons OR muscle OR soft tissue OR knee OR ankle OR foot OR shoulder OR hip)) AND (injection)) AND (("2020/02/24"[Date - Publication] : "3000"[Date - Publication]))	celestone OR corticosteroid)

24. (soft tissue or soft-tissue).ti,ab.<br><br>25. sprains.ti,ab.<br><br>26. strains.ti,ab.<br><br>27. exp "Sprains and Strains"/<br><br>28. contusion.ti,ab. or exp Contusions/<br><br>29. exp Tendinopathy/ or tendin*.ti,ab.<br><br>30. bursitis.ti,ab. or exp Bursitis/<br><br>31. (tear* or rupture*).ti,ab.<br><br>32. impingement*.ti,ab.<br><br>33. 21 or 22 or 23 or 24 or 25 or 26 or 27 or 28 or 29 or 30 or 31 or 32<br><br>34. 10 and 20 and 33<br><br>35. limit 34 to humans	22. exp Tendon/ or tendon*.ti,ab.<br><br>23. muscle.ti,ab. or exp Muscle/<br><br>24. (soft tissue or soft-tissue).ti,ab.<br><br>25. sprain.ti,ab. or exp sprain/<br><br>26. muscle strain.ti,ab. or exp muscle strain/<br><br>27. contusion.ti,ab. or exp Contusion/<br><br>28. exp Tendinitis/ or tendin*.ti,ab.<br><br>29. bursitis.ti,ab. or exp Bursitis/<br><br>30. (tear or rupture).ti,ab.<br><br>31. impingement*.ti,ab.<br><br>32. 21 or 22 or 23 or 24 or 25 or 26 or 27 or 28 or 29 or 30<br><br>33. 10 and 20 and 32<br><br>34. limit 33 to humans		

Appendix 2: Functional Outcomes <6 weeks, Constant Scores, Mean Difference (MD)

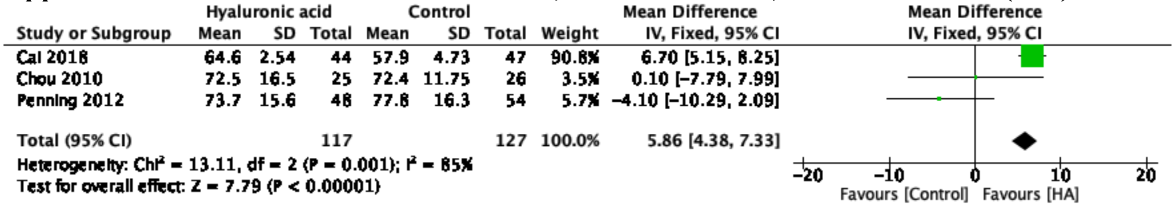

Appendix 3: Functional Outcomes <6 weeks, Standardized Mean Difference

Study or Subgroup	HA Mean	HA SD	HA Total	Control Mean	Control SD	Control Total	Weight	Std. Mean Difference IV, Fixed, 95% CI
Cai 2018	64.6	2.54	44	57.9	4.73	47	21.5%	1.73 [1.25, 2.22]
Chou 2010	72.5	16.5	25	72.4	11.75	26	16.8%	0.01 [−0.54, 0.56]
Kim 2012	21.6	6	38	21.6	4.5	42	26.3%	0.00 [−0.44, 0.44]
Merolla 2013	69.7	1.16	25	59.2	1.88	23	2.2%	6.68 [5.17, 8.18]
Penning 2012	73.7	15.6	48	77.8	16.3	54	33.2%	−0.25 [−0.65, 0.14]
Total (95% CI)			180			192	100.0%	0.44 [0.21, 0.66]

Heterogeneity: $Chi^2 = 111.74$, $df = 4$ ($P < 0.00001$); $I^2 = 96\%$
Test for overall effect: $Z = 3.82$ ($P = 0.0001$)

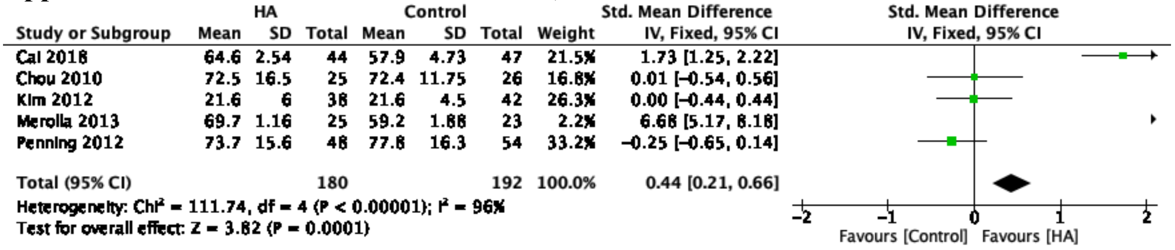

Appendix 4: HA vs. Saline – all indications - < 6 weeks, VAS, Mean Difference

Study or Subgroup	HA Mean	HA SD	HA Total	Control Mean	Control SD	Control Total	Weight	Mean Difference IV, Fixed, 95% CI
Cai 2018	4.8	1.32	44	5.62	0.99	47	14.6%	−0.82 [−1.30, −0.34]
Chou 2010	4.2	1.76	25	4.77	1.75	26	3.7%	−0.57 [−1.53, 0.39]
Huang 2018	3	2.22	18	3	1.48	9	1.7%	0.00 [−1.41, 1.41]
Ozgen 2010	0.5	2.96	12	0	2.22	12	0.8%	0.50 [−1.59, 2.59]
Penning 2012	5.4	2.54	44	4.4	2.65	48	3.0%	1.00 [−0.06, 2.06]
Petrella 2007	−4.04	1.16	77	−2.42	1.09	75	26.6%	−1.62 [−1.98, −1.26]
Petrella 2010	2.2	1.2	165	7.1	1.3	166	46.8%	−4.90 [−5.17, −4.63]
Rezasoltani 2021	3.6	1.75	28	6.5	2.2	23	2.8%	−2.90 [−4.01, −1.79]
Total (95% CI)			413			406	100.0%	−2.91 [−3.10, −2.73]

Heterogeneity: $Chi^2 = 433.06$, $df = 7$ ($P < 0.00001$); $I^2 = 98\%$
Test for overall effect: $Z = 30.95$ ($P < 0.00001$)

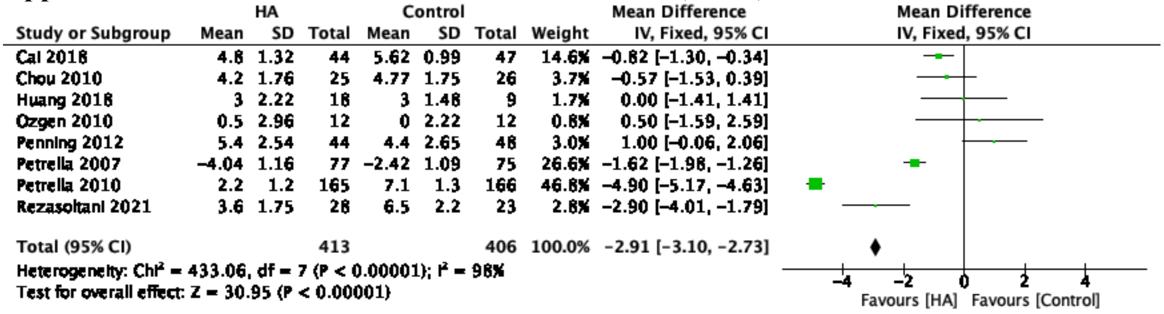

Appendix 5: Funnel Plot

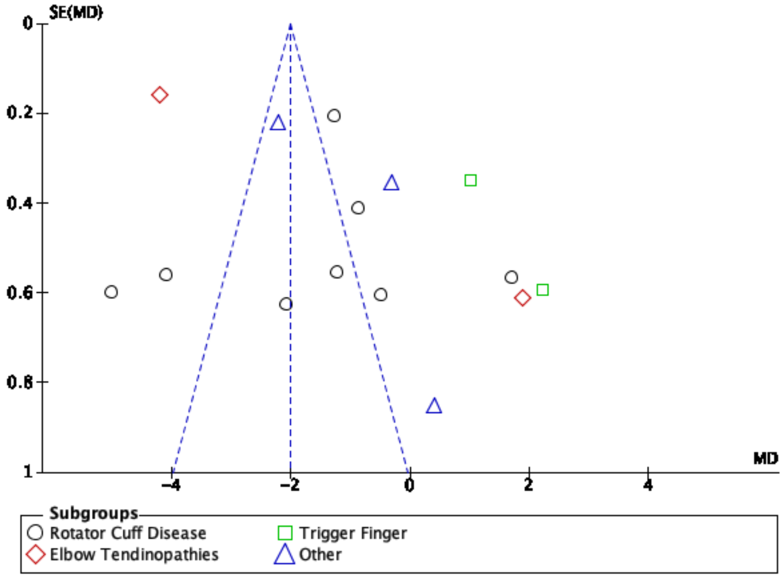

Appendix 6: Pain > 12 weeks, Mean Difference, VAS Score

Study or Subgroup	Hyaluronic acid Mean	SD	Total	Control Mean	SD	Total	Weight	Mean Difference IV, Fixed, 95% CI
**1.3.1 Rotator Cuff Disease**								
Cai 2018	3.45	1.09	44	5.85	1.27	47	19.1%	−2.40 [−2.89, −1.91]
Penning 2012	4.3	3.05	44	3.6	3.18	48	2.8%	0.70 [−0.57, 1.97]
Shibata 2001	3.4	1.2	13	3.9	1.6	13	3.8%	−0.50 [−1.59, 0.59]
Subtotal (95% CI)			101			108	25.6%	−1.78 [−2.20, −1.36]
Heterogeneity: Chi² = 26.16, df = 2 (P < 0.00001); I² = 92%								
Test for overall effect: Z = 8.35 (P < 0.00001)								
**1.3.2 Other**								
Kanchanathepsak 2020	1	1.75	26	0	2.5	31	3.7%	1.00 [−0.11, 2.11]
Lynen 2016	0.3	0.15	29	2.2	1.4	30	17.7%	−1.90 [−2.40, −1.40]
Petrella 2010	2.4	1.4	165	7.7	1.3	166	53.0%	−5.30 [−5.59, −5.01]
Subtotal (95% CI)			220			227	74.4%	−4.18 [−4.43, −3.94]
Heterogeneity: Chi² = 219.51, df = 2 (P < 0.00001); I² = 99%								
Test for overall effect: Z = 33.34 (P < 0.00001)								
**Total (95% CI)**			321			335	100.0%	−3.57 [−3.78, −3.35]
Heterogeneity: Chi² = 339.40, df = 5 (P < 0.00001); I² = 99%								
Test for overall effect: Z = 32.98 (P < 0.00001)								
Test for subgroup differences: Chi² = 93.73, df = 1 (P < 0.00001), I² = 98.9%								

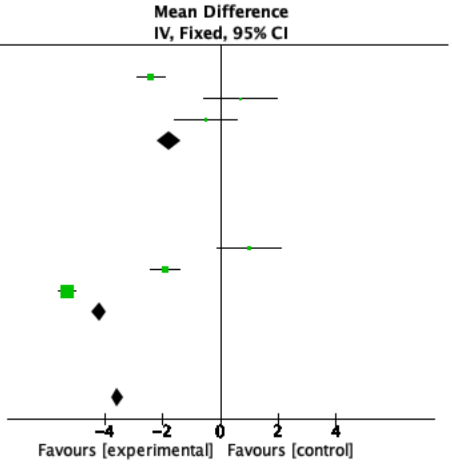

Appendix 7: Functional Outcomes >6 weeks, </=12 weeks Constant Scores, Mean Difference (MD

Study or Subgroup	Hyaluronic acid Mean	SD	Total	Control Mean	SD	Total	Weight	Mean Difference IV, Fixed, 95% CI
Cai 2018	72.3	2.56	44	58.1	3.25	47	22.6%	14.20 [13.00, 15.40]
Chou 2010	79.2	13.1	25	69.1	13.3	26	0.6%	10.10 [2.85, 17.35]
Merolla 2013	67.4	1.09	25	59.3	1.21	23	76.0%	8.10 [7.45, 8.75]
Penning 2012	74.5	15.9	47	78.2	16.9	53	0.8%	−3.70 [−10.13, 2.73]
**Total (95% CI)**			141			149	100.0%	9.40 [8.83, 9.97]
Heterogeneity: Chi² = 92.83, df = 3 (P < 0.00001); I² = 97%								
Test for overall effect: Z = 32.34 (P < 0.00001)								

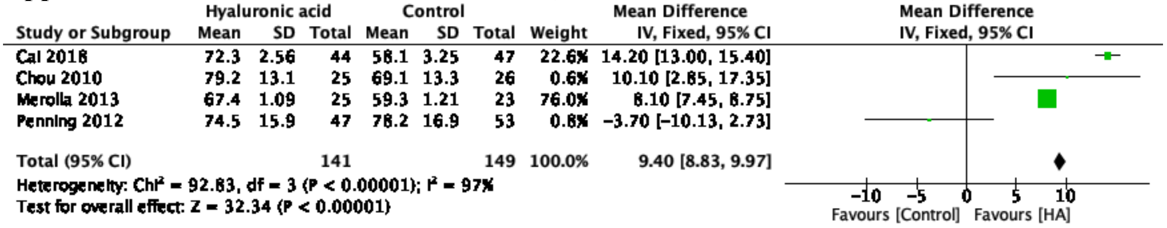

Supplement: sj-pdf-1-sph-10.1177_19417381211073316 – Supplemental material for The Role of Hyaluronic Acid for Soft Tissue Indications: A Systematic Review and Meta-Analysis [file sj-pdf-1-sph-10.1177_19417381211073316.pdf]
